# Ensembl 2014

**DOI:** 10.1093/nar/gkt1196

**Published:** 2013-12-06

**Authors:** Paul Flicek, M. Ridwan Amode, Daniel Barrell, Kathryn Beal, Konstantinos Billis, Simon Brent, Denise Carvalho-Silva, Peter Clapham, Guy Coates, Stephen Fitzgerald, Laurent Gil, Carlos García Girón, Leo Gordon, Thibaut Hourlier, Sarah Hunt, Nathan Johnson, Thomas Juettemann, Andreas K. Kähäri, Stephen Keenan, Eugene Kulesha, Fergal J. Martin, Thomas Maurel, William M. McLaren, Daniel N. Murphy, Rishi Nag, Bert Overduin, Miguel Pignatelli, Bethan Pritchard, Emily Pritchard, Harpreet S. Riat, Magali Ruffier, Daniel Sheppard, Kieron Taylor, Anja Thormann, Stephen J. Trevanion, Alessandro Vullo, Steven P. Wilder, Mark Wilson, Amonida Zadissa, Bronwen L. Aken, Ewan Birney, Fiona Cunningham, Jennifer Harrow, Javier Herrero, Tim J.P. Hubbard, Rhoda Kinsella, Matthieu Muffato, Anne Parker, Giulietta Spudich, Andy Yates, Daniel R. Zerbino, Stephen M.J. Searle

**Affiliations:** ^1^European Molecular Biology Laboratory, European Bioinformatics Institute, Wellcome Trust Genome Campus, Hinxton, Cambridge, CB10 1SD and ^2^Wellcome Trust Sanger Institute, Wellcome Trust Genome Campus, Hinxton, Cambridge, CB10 1SA, UK

## Abstract

Ensembl (http://www.ensembl.org) creates tools and data resources to facilitate genomic analysis in chordate species with an emphasis on human, major vertebrate model organisms and farm animals. Over the past year we have increased the number of species that we support to 77 and expanded our genome browser with a new scrollable overview and improved variation and phenotype views. We also report updates to our core datasets and improvements to our gene homology relationships from the addition of new species. Our REST service has been extended with additional support for comparative genomics and ontology information. Finally, we provide updated information about our methods for data access and resources for user training.

## INTRODUCTION

The Ensembl project (http://www.ensembl.org) creates and distributes genome annotations and provides integrated views of other valuable genomic data for supported chordate genomes. Our resources are intended to serve as community reference datasets on which other genomic research can be built. As such, Ensembl provides unique tools, datasets and user support compared to similar projects such as the UCSC Genome Browser (1), while supporting community standards that promote interoperability in genomics. For example, we have developed and distribute an extensive, open software infrastructure with diverse analysis pipelines supporting a variety of genome analyses (2) and the artificial intelligence inspired eHive analysis management system (3); data mining and analysis tools that include BioMart (4) and the Ensembl Variant Effect Predictor (VEP) (5); supported and robust application programing interfaces (APIs) (6) and a unique genome browser interface (7). Our software is distributed using a permissive Apache-style open-source license meaning that, unlike similar software, it is free for all potential users. Additionally, our data is provided without restriction and we have the most comprehensive suite of training options of any public genomics tool to maximize usability. In common with the UCSC Genome Browser, Ensembl supports community standard file formats such as BAM, BED, wiggle and other common file types. We have also incorporated support for track hubs over the past year to enable researchers to set up and view large-scale datasets. For example, the data produced by the ENCODE consortium (8) can be viewed by loading the ENCODE track hub (9) and users can then access an experiment matrix within the Ensembl configuration menu and quickly select datasets by cell or experiment type. However, over and above simply displaying these data, Ensembl uses them as described below in our integrative Regulatory Build analysis resulting in an evidence-based annotation of whole-genome regulation.

Ensembl resources are available for a total of 77 species as of release 73 (September 2013) with human, mouse, zebrafish, rat and various farm animals having the most extensive support. For 60 chordate species, we have full support comprising evidence-based gene annotation and comparative genomics analysis. In addition, for 18 of these species, there are variation resources and regulatory annotation for human and mouse. At present 13 additional chordate species are accessible with basic support via Ensembl preview sites (available from http://pre.ensembl.org), which provide BLAST access to the genome data and genome visualization, but not a complete gene build. Three non-chordate model species are also fully supported by Ensembl—worm (*Caenorhabditis elegans*), fruit fly (*Drosophila melanogaster*) and yeast (*Saccharomyces cerevisiae*)—with imported annotation from their respective genome databases in partnership with the Ensembl Genomes project (10). All fully supported species are accessible via the Ensembl BioMart, the Ensembl APIs and web displays. All data are also available for querying via our public MySQL servers, as full data downloads and as an Amazon public dataset.

Since our last report (11), we have added two new species with full gene annotation and comparative genomics support: duck (*Anas platyrhynchos*) (12) and collared flycatcher (*Ficedula albicollis*) and one new species with variation support: gibbon. New assemblies with corresponding updates to the gene annotations, alignments and variation data were also provided for rat, cat and chicken. At the same time, we added seven new species with basic support on the Ensembl preview site: blind cave fish (*Astyanax mexicanus*), white rhinoceros (*Ceratotherium simum simum*), baboon (*Papio anubis*), prairie vole (*Microtus ochrogaster*), vervet monkey (*Chlorocebus sabaeus*), naked mole-rat (*Heterocephalus glaber*) and aardvark (*Orycteropus afer*) and updated the preview sites for common shrew (*Sorex araneus*), bottle-nosed dolphin (*Tursiops truncatus*), American pika (*Ochotona princeps*) and armadillo (*Dasypus novemcinctus*) with new assemblies. In addition, as the human and mouse genome assemblies are updated regularly by the Genome Reference Consortium (GRC) to include alternate sequences in the form of ‘fix’ and ‘novel’ assembly patches (13), we include these additional alternate sequences and annotate them with genes, variation and other features as appropriate. Ensembl release 73 (September 2013) included the human GRCh37.p12 assembly (i.e. the twelfth patch release of the GRCh37 assembly) and the GRCm38.p1 mouse assembly.

In addition to the newly support species and community standards reported above, the most important updates over the last year that have advanced the project since our last report (11) include new and more comprehensive phenotype annotations most valuable to those interested in human disease research, scrollable genome browsing designed to appeal to all users of our web interface and new REST endpoints supporting more flexible analysis options for those users that interact with the Ensembl resources programmatically. These and other features are described in more detail below.

### Ensembl browser

**Figure 1.**
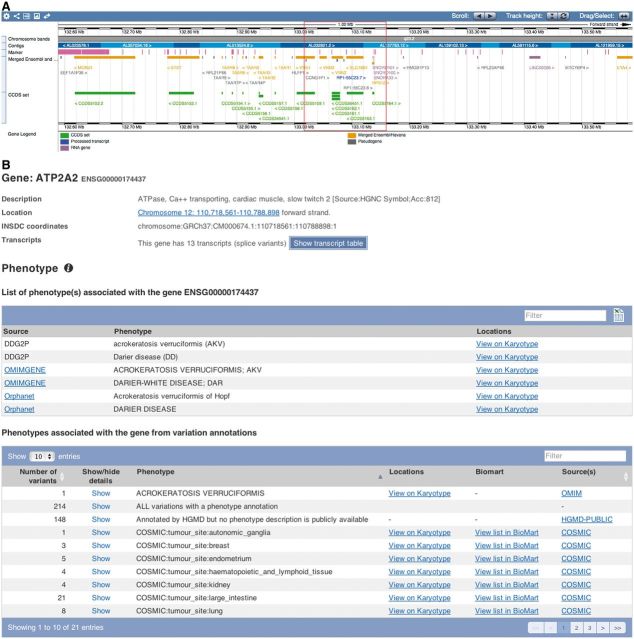
(**A**) The scrollable Genoverse view (with view control icons in the upper right corner) provides the overview panel on the Region in Detail page. Image from URL: http://e73.ensembl.org/Homo_sapiens/Location/View?r=6:133017695-133161157. (**B**) Phenotype data from DDG2P, OMIM, Orphanet, HGMD and COSMIC for the human gene ATP2A2. Image created from URL http://e73.ensembl.org/Homo_sapiens/Gene/Phenotype?db=core;g=ENSG00000174437;r=12:110718561-110788898.

This year we significantly updated Ensembl’s main Region in Detail page with the full incorporation of the Javascript-based, scrollable and zoomable browser, Genoverse, in place of the overview panel that had been a part of Ensembl for >10 years. Older, unsupported browsers fall back to the previous non-scrolling overview image. Genoverse allows users to scroll back and forth along the genome and update the main image below it to show the new region (Figure 1A). Our search engine was also upgraded from Lucene to Solr and we implemented a new search interface with features such as faceting and auto-completion.

Our web displays dedicated to variation and phenotype data were also markedly improved. We specifically focused on displays for structural variants, which are now coloured by class and the higher quality structural variants from the 1000 Genomes Project are provided in a separate track. We introduced a page for structural variant phenotype data and now have additional phenotype data, including the variants from NCBI’s ClinVar project that are classified as being probable-pathogenic, pathogenic, drug-response or histocompatibility. Phenotype data from multiple sources are integrated and displayed for relevant genes (Figure 1B). Variants in regulatory regions are now annotated on all tracks and variant names are visible when zoomed in on all displays. We have also improved the VEP visual output in the form of summary pie charts.

Beyond these major developments, there have been other important improvements. In particular, we improved the handling of user data with a streamlined upload interface and support for uploading VEP output files. Additionally, configuration of complex data hubs has been made easier by displaying the track options in a matrix similar to the existing configurations for regulation data.

### Ensembl annotations

All Ensembl annotations whether gene, variation or regulation, are based on integration of relevant data sources. We update the human gene set for every Ensembl release via a merge of the Ensembl evidence-based automatic annotation and Havana (14) manual annotation to produce an updated GENCODE gene set. This set also includes all current human Consensus Coding Sequence (CCDS) gene models (15). Manual annotation from Havana is additionally incorporated into our gene sets on alternate releases for zebrafish (16) and for mouse, which also includes all current CCDS gene models. Pig includes manual annotation from Havana on selected regions of the genome. The year 2013 has seen the inclusion of RNASeq data for seven species: human, chicken, cat, collared flycatcher, gibbon, rabbit and anole lizard. For gibbon, rabbit and anole lizard, the RNASeq data were used to update an existing standard gene annotation whereas for other species (except human) the data were integrated into the annotation as part of the primary gene-build process. Some of these species are provided with tissue-specific RNASeq samples which allow users to explore tissue-specific expression.

Ensembl variation annotation integrates all publicly available variation datasets to provide a coherent and complete resource for variome interpretation across 217 million variants in the 18 Ensembl species with supported variation resources. Basic variation data including genomic location, allele changes, allele and genotype frequencies and population data are imported for SNPs and indels from dbSNP (17) and for structural variants from DGVa (18). Additional human data are imported directly from the 1000 Genomes Project (19), the Exome Sequencing Project (20) and from 14 individual genomes that provide genotype information. In addition to the newly supported gibbon data listed above, over the past year, updated variation resources were released for human, platypus, cow, mouse, pig, zebrafish, opossum, orang-utan and macaque. We also extended cross-references to new and popular genotyping chips for human, chicken, horse and cow.

We significantly expanded our support for human phenotype data in Ensembl beyond the UniProt (21), OMIM (22), EGA, HGMD Public (23) (variation location only), COSMIC somatic mutations (24) and NHGRI GWAS Catalog (25) resources that we have supported in the past. New data from ClinVar, Orphanet (26), the Developmental Disorder Genotype—Phenotype Database (DDG2P) from DECIPHER (27), dbGaP, Phencode and the MAGIC and GIANT consortiums have now been fully integrated into Ensembl (Figure 1B). From these data sources, we select only the significant associations to display on the website and provide full datasets in the database and BioMart. For variants stored in LOVD (28), Ensembl queries LOVD directly and displays the information on the appropriate variation web page. In addition, we have now incorporated phenotype information for other species. Mouse phenotype data are provided from international projects including EuroPhenome (29) and IMPC (International Mouse Phenotyping Consortium) (30). For other animals, data are imported from the Online Mendelian Inheritance in Animals (OMIA) database (31). This year we have also developed a new pipeline to cross-reference publications citing variants from EuropePMC, NCBI and UCSC.

Regulatory annotation in Ensembl is currently available across multiple human and mouse cell lines. The main resource, the Ensembl Regulatory Build is a comprehensive synthesis of functional assays provide by a number of consortia, such as ENCODE (8) and Roadmap Epigenomics Mapping Consortium (32). Although the raw data from these projects can be displayed directly on Ensembl through dedicated track hubs, the Regulatory Build is a higher level integrated analysis that defines a collection of Regulatory Features (i.e. regions of the genome that display regulatory activity in one of 13 human or five murine cell lines). Where relevant, transcription-factor binding sites are predicted on these regions using the JASPAR binding motifs (33).

Additionally, Ensembl links out to relevant externally curated databases of regulatory data including enhancer regions from VISTA (34), miRNA binding sites from Microcosm http://www.ebi.ac.uk/enright-srv/microcosm/) and eQTLs from Genevar (35). Several reference DNA methylation experiments are also included.

## COMPARATIVE GENOMICS RESOURCES

Whole-genome alignments of vertebrate species are provided within the Ensembl Compara database. Because all genome assemblies are not sequenced to the same level of completeness, we group the assemblies into two tiers for differential processing. High quality genomes from 13 species are aligned into a progressive multiple sequence alignment using the EPO (Enredo-Pecan-Ortheus) pipeline (36,37), which also estimates the underlying ancestral genome sequences. The low coverage genomes of an additional 23 species, that are much more fragmented, are inserted into the previous alignment by mapping them onto the human assembly with LASTZ. We also produce clade-specific multiple sequence alignments for primates, birds, fish and amniotes. In particular, the fish multiple alignment has been extended to eight species this year. From these alignments, we compute the conservation at every position, using GERP (38).

Our gene-based comparative genomics resources are updated every release to incorporate new species, updated assemblies and gene annotation sets. These include gene phylogenetic trees, gene families and gene dynamics. This year, the inclusion of duck and collared flycatcher and an update of the guide species tree have greatly improved the quality of the gene trees in the Sauria clade and reduced the number of poorly supported duplications from 74% to 30%. In close collaboration with the TreeFam (39) and Ensembl Genomes (10) projects, we will migrate to an HMM-based classification for GreeTree annotation, which will reduce a key quadratic complexity to a linear one.

### Data access, data mining and quality control

**Figure 2.**
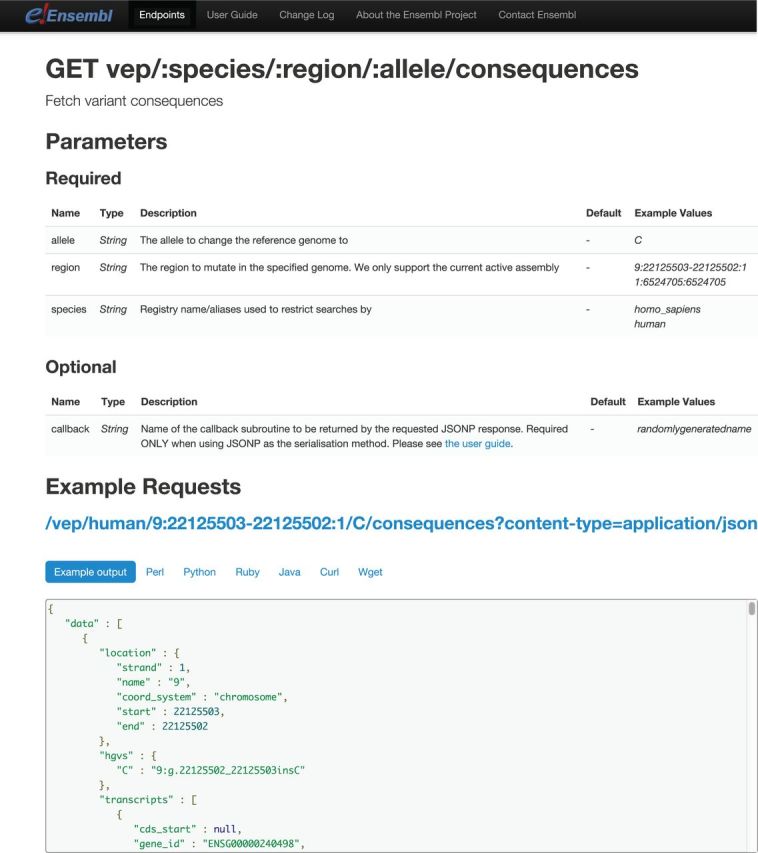
Usage and example output for the Ensembl REST server Fetch Variant Consequences endpoint.

Ensembl’s REST service, available at http://beta.rest.ensembl.org, continues to be actively developed as a public beta (Figure 2). This year has seen the addition of 12 new endpoints including access to translation features, SNVs and protein domains, as well as access to whole-genome alignments by region. Additionally, the REST API is now able to query GeneTrees by their containing member stable identifier or gene symbols such as HGNC. Other new endpoints include the ability to use a stable identifier to identify overlapping features and location information as well as access to NCBI taxonomy and ontology datasets. The ontology endpoints currently provide the gene ontology (GO) (40), sequence ontology (SO) (41) and experimental factor ontology (EFO) (42) information used within Ensembl. The REST service will move out of beta during the next year coinciding with the introduction of POST requests and improved VEP integration.

The more established BioMart data-mining tool (43) provides users with a variety of ways of accessing the Ensembl data quickly and with relative ease. Users can choose to access the data via the MartView web interface or via the MartService routes including the BioMart Perl API, DAS server, SOAP, REST or BioConductor biomaRt package. The Ensembl BioMart databases (4) are built from scratch each release in order to incorporate the latest annotated and imported data, and they are current with the data resources described above.

Beyond programmatic and tool-based data-access methods, we continue to provide complete data downloads in a variety of formats including the VCF files that were introduced this year to distribute many subsets of the Ensembl variation data.

To manage the increasing size and complexity of Ensembl releases, we have increased our quality control (QC) procedures over the past year. These are an essential part of each release cycle and range from validation testing of the various APIs to methods for checking data and database integrity. The Ensembl gene set is also independently analyzed using a specific curation/QC pipeline run for all updates of the human and mouse gene sets. This procedure compares the set of Ensembl translations for a particular species directly to the publicly available sequence resources UniProt (21) and RefSeq (44) and reports the percentage identity of the alignments. In addition to the above species, the pipeline has been employed for the rat, zebrafish and chicken genomes.

Ensembl variation and regulatory resources also rely on comprehensive and flexible infrastructure to manage the growing amount of relevant datasets in the public domain. In preparation for the updated human reference genome (GRCh38) expected at the end of 2013, we consolidated these pipelines to work automatically over a large number of files with minimal supervision. Specifically, more of our pipelines are run using the eHive system (3) and employ both a modular structure to the analysis and on-the-fly calculation for specific data types stored in the databases.

### Outreach and training

Ensembl supports users through worldwide face-to-face training workshops, our helpdesk@ensembl.org and dev@ensembl.org email lists, online training and social media. This year we made several changes to intensify and connect our user interactions on Twitter (https://twitter.com/Ensembl), Facebook (https://www.facebook.com/Ensembl.org) and the Ensembl Blog (http://www.ensembl.info/). Together these methods have proven extremely effective for supporting users beyond the traditional mailing list and FAQ model that we continue to maintain.

Distance and on-line training are provided through our Helpdesk channel on YouTube, which saw >70% growth in the number of subscribers and incorporated two new videos during the year. For those users wanting more intensive training, we filmed and now provide a one-day Ensembl browser workshop (http://www.ebi.ac.uk/training/online/course/ensembl-filmed-browser-workshop) and a three-day Ensembl API workshop (http://www.ebi.ac.uk/training/online/course/ensembl-filmed-api-workshop), both complete with videos and exercises. A Quick Ensembl course providing a short introduction to the browser (http://www.ebi.ac.uk/training/online/course/ensembl-quick-tour-0) complements the more complete workshops.
